# Spotting the clues: cluster of differentiation—a perspective of immune response intertwined with dysautonomia in colon cancer – a prospective cohort study

**DOI:** 10.25122/jml-2025-0014

**Published:** 2025-02

**Authors:** Diana-Theodora Morgos, Lucian-George Eftimie, Horia Nicolae, Remus Iulian Nica, Constantin Stefani, Daniela Miricescu, Adrian Tulin, Florin Mihail Filipoiu

**Affiliations:** 1 Doctoral School, Discipline of Anatomy, Carol Davila University of Medicine and Pharmacy, Bucharest, Romania; 2 Discipline of Anatomy and Biomechanics, Faculty of Physical Therapy, National University of Physical Education and Sports, Bucharest, Romania; 3 Dr. Carol Davila Central Military Emergency University Hospital, Bucharest, Romania; 4 Discipline of Neurology, Faculty of Medicine, Carol Davila University of Medicine and Pharmacy, Bucharest, Romania; 5 Discipline of Neurology, Elias University Emergency Hospital, Bucharest, Romania; 6 Discipline of General Surgery, Faculty of Medicine, Carol Davila University of Medicine and Pharmacy, Bucharest, Romania; 7 Department I of Family Medicine and Clinical Base, Dr. Carol Davila Central Military Emergency University Hospital, Romania; 8 Discipline of Biochemistry, Faculty of Dentistry, Carol Davila University of Medicine and Pharmacy, Bucharest, Romania; 9 Discipline of Anatomy, Carol Davila University of Medicine and Pharmacy, Bucharest, Romania

**Keywords:** dysautonomia, colon cancer, immunohistochemistry, cluster of differentiation

## Abstract

Dysautonomia, a parasympathetic-sympathetic imbalance, has clinical and public health consequences. Colon neoplasm is linked to dysautonomia through a complex interplay between the two conditions. In this prospective cohort study, we evaluated 18 patients divided into three groups: six patients with both colon cancer and dysautonomia, six patients with colon cancer without dysautonomia, and six patients with dysautonomia only (control group). Dysautonomia was defined by the presence of orthostatic hypotension, a non-increased or dropped heart rate, and various autonomic symptoms. During abdominal surgery, tissue samples from the celiac ganglion were collected and analyzed using immunohistochemistry (IHC). Our findings revealed a significant correlation between IHC marker expression in colon cancer and dysautonomia (control) (r = 0.927, *P* = 0.008). ANOVA results confirmed that the model was significant and that the dysautonomia group (control) had a significant effect on the independent variables (colon cancer or colon cancer + dysautonomia). The study proposes that a shared immunological mechanism underlies both dysautonomia and colon cancer, suggesting that the immune system plays a crucial role in the development or progression of these two conditions.

## INTRODUCTION

Colon cancer is the third most common type of cancer worldwide and the second deadliest malignancy for both sexes combined [1]. Its incidence increases with age, with most cases occurring in individuals over 50; however, recent studies indicate a worrisome trend of rising rates among younger adults [2]. Colon cancer originates in the epithelial cells of the colon and rectum, frequently starting as benign polyps that can evolve into malignant tumors over time. It ranks as the third most common cancer globally and is a significant cause of cancer-related deaths, influenced by a mix of genetic, environmental, and lifestyle factors. Risk factors include age, family history, dietary choices, lack of physical activity, and genetic conditions such as Lynch syndrome and familial adenomatous polyposis. The pathophysiology of the cancer involves an intricate series of molecular changes, including mutations in oncogenes and tumor suppressor genes, that drive tumor formation [3].

Paraneoplastic autonomic dysfunction can be categorized as immune-mediated or nonimmune-mediated, and in patients displaying symptoms like orthostatic hypotension, a paraneoplastic disorder should be suspected [4]. Autonomic dysfunction, or dysautonomia, can appear before, during, or after cancer treatment, with orthostatic hypotension potentially becoming so severe that it confines patients to bed despite efforts to manage their blood pressure. This form of autonomic dysfunction has been observed across various cancers, including small-cell lung cancer, other lung tumors, thymoma, as well as ovarian and breast cancers, and can sometimes manifest in the absence of detectable cancer [5]. For individuals without a cancer diagnosis, symptoms coupled with clinical signs of an underlying tumor, such as unexplained weight loss or risk factors like heavy smoking and family cancer history, may indicate a connection between autonomic symptoms and an undiagnosed malignancy. Furthermore, the presence of colon cancer is associated with alterations in autonomic function, particularly a shift in the balance between parasympathetic and sympathetic responses, characterized by heightened sympathetic activity, which may disrupt gastrointestinal motility [6].

Symptoms of dysautonomia include postural orthostatic tachycardia, orthostatic hypotension—one of its most characteristic features—dizziness or lightheadedness, sweating abnormalities, digestive issues such as constipation or diarrhea, urinary retention, sensations of warmth, and nausea [7]. We decided to perform a prospective cohort study to determine the correlations between dysautonomia and colon cancer. Dysautonomia is commonly associated with various diseases, such as type 2 diabetes, Parkinson’s disease, amyloidosis, amyotrophic lateral sclerosis, and autoimmune autonomic ganglionopathy. However, its potential connection to colon cancer has not been explored. This study aimed to investigate the presence of immune cells in the sympathetic ganglia of individuals with dysautonomia and colon cancer, given the well-established role of the immune system in oncogenesis. Our research aimed to determine whether dysautonomia can significantly impact the immune response to colon cancer.

## MATERIAL AND METHODS

This prospective cohort study was conducted at the Dr. Carol Davila Central Military Emergency Hospital, Bucharest, Romania, between May 2023 and May 2024. Our cohort included 18 patients, divided into three groups: six patients with colon cancer and dysautonomia, six patients with colon cancer without dysautonomia, and six patients with dysautonomia alone (the control group). Patients were considered eligible if they were diagnosed with colon cancer and underwent surgery for this pathology or if they had been previously diagnosed with dysautonomia and underwent a laparotomy for benign conditions, including a colectomy (duodenal ulcer perforation, cholecystectomy). Patients with dysautonomia and cancer-free were considered the control group. Patients had to be at least 18 years old to be enrolled, and signed informed consent was mandatory for study participation. Patients were excluded in case of ongoing pregnancy or any associated medical history that would interfere with the study protocol.

Dysautonomia was diagnosed based on the presence of significant orthostatic hypotension, defined as a blood pressure drop of at least 20/10 mmHg within 3 minutes of standing, a non-increased or dropped heart rate within the same timeframe, and symptoms such as sweating abnormalities, warmth, and nausea. Patients meeting all three criteria were classified as having dysautonomia, regardless of colon cancer status. Patients with no history of colon cancer but diagnosed with dysautonomia were included as the control group.

The study protocol involved a baseline pre-operative evaluation consisting of a physical examination to confirm or rule out dysautonomia. An orthostatic test was performed to assess the diagnostic criteria. During surgery, celiac ganglia tissue samples were collected from all patients. These tissue specimens, obtained intraoperatively, were immediately processed for immunohistochemistry (IHC) analysis. We studied the celiac ganglion, a representative sympathetic ganglion readily excised during laparotomy surgery. Statistical analysis was conducted to determine potential correlations between colon cancer and dysautonomia.

The primary outcome measures were the analysis of tissue samples from celiac ganglia to identify potential IHC markers associated with dysautonomia and colon cancer. The study aimed to investigate whether the expression of specific immune markers (CD20, CD5, CD8, CD68, CD23, and CD45) was detectable in the celiac ganglia of patients with colon cancer, those with colon cancer and concomitant dysautonomia, and patients with isolated dysautonomia. The presence of these markers across all groups may suggest a shared pathway between colon cancer and dysautonomia that involves the immune system.

### Sample processing protocol

A standard histological examination was conducted utilizing hematoxylin and eosin (H&E) staining and additional IHC testing, which is essential for an accurate diagnosis. The preparation of tissue samples involved several steps: collecting the tissue, fixing it in a 10% formalin to preserve its structure and prevent degradation, dehydrating the tissue by progressively increasing ethanol concentrations to remove water and prevent autolysis, clearing the tissue using xylene to remove the dehydrating agent, preparing the tissue for embedding by placing it in paraffin wax, and finally, creating a solid block that could be sectioned for examination.

Tissue sections were cut to a thickness of 3-5 μm using a microtome, with the sectioning orientation carefully chosen to ensure that the desired structures were visible and accessible for staining. Tissue sections were mounted onto glass slides using a suitable mounting medium and thoroughly dried. Antigen retrieval was performed through heat-induced epitope retrieval (HIER) to expose antigenic sites for staining.

The staining process involved applying a primary antibody specific to the target antigen on the tissue section, followed by an enzyme-conjugated secondary antibody detection system to visualize the target antigen. Hematoxylin was used for counterstaining, enhancing contrast, and improving visualization of the target antigen. Finally, to protect the stained tissue and ensure even light transmission, a mounting medium was used to seal the slide, and a coverslip was placed over the mounted slide to prevent damage.

Microphotographs of the H&E-stained sections were taken for reference using a classic optical microscope with bright field (Carl Zeiss Axio Imager.M2p) equipped with an Axiocam 305 color digital camera (Carl Zeiss Microscopy GmbH) at the Pathology Department of the Dr. Carol Davila Central Military Emergency University Hospital in Bucharest. Both low and high magnifications, ranging from 50x to 200x, were used. We tested 108 histological samples, including 6 samples from each of the 18 patients, each with celiac ganglion tissue stained with one of the IHC markers. Table 1 provides the characteristics of the primary antibodies used for IHC.

**Table 1:** Primary antibodies of IHC

Antibody	Clone name	Host	Clonality	Company	Catalog No.	RRID	Dilution
CD20	L26	mouse	monoclonal	Ventana Medical Systems	760-2531	AB_2335956	1:100
CD5	SP19	rabbit	monoclonal	Ventana	790-4451	AB_2335984	1:100
CD8	SP57	rabbit	monoclonal	Ventana	790-4460	AB_2335985	1:100
CD68	KP-1	mouse	monoclonal	Ventana	790-2931	AB_2335972	1:100
CD23	SP23	rabbit	monoclonal	Ventana	790-4408	AB_2336017	1:100
CD45	LCA (RP2/18)	mouse	monoclonal	Ventana	760-2505	AB_2335953	1:100

### Statistical analysis

Statistical analysis was performed using SPSS 26.0 for Windows (SPSS Inc., Chicago, IL, USA) and Microsoft Excel 2019. Descriptive statistics were used to summarize demographic and clinical characteristics of the study population. Inferential statistics (i.e., *t*-tests, ANOVA, Pearson correlations test, Kendall’s tau, and Spearman’s rho test) was used to compare means and proportions between groups (colon cancer with dysautonomia, colon cancer without dysautonomia, and the control group) for continuous and categorical variables, respectively. Logistic regression analysis was used to estimate the probability of dysautonomia in patients with colon cancer, with dysautonomia as the dependent variable.

## RESULTS

The study cohort consisted of 18 patients, including eight women (44.4%) and ten men (55.5%) with a median age of 47.5 years at inclusion. The standard deviation of the age was 2.23, with a variance of 4.99. The analyzed variables were continuous and had a normal distribution. Table 2 shows the demographic and clinical characteristics of the cohort.

**Table 2:** Demographic and clinical characteristics of the cohort

Variable	Median (Range) /No. of patients (%)
Age	47.5 (40-55)
Gender	
Men	10 (55.5%)
Women	8 (44.4%)
Patients with dysautonomia	6 (33.3%)
Patients with colon cancer	6 (33.3%)
Patients with colon cancer and dysautonomia	6 (33.3%)

A total of 108 histological samples were analyzed. For each of the 18 patients, the expression of the IHC markers CD20, CD5, CD8, CD68, CD23, and CD45 was assessed on tissue samples of celiac ganglia. The mean values for IHC marker expression were calculated for each patient category, and the results are summarized in Table 3. Each expression of the IHC marker was quantified as a percentage of the total number of samples tested, clearly representing the prevalence of positive staining for each marker across the samples analyzed. This method allows for straightforward comparisons between different markers and enhances the interpretability of the data. Table 3 reveals the percentage of positive-stained celiac ganglia samples with specific IHC antibodies.

**Table 3:** Percentage of positive-stained celiac ganglia samples

Group (*n* = 36 samples per group)	CD 20	CD 5	CD 8	CD 68	CD 23	CD 45
Dysautonomia (control)	90%	80%	45%	40%	35%	99%
Colon cancer	99%	95%	60%	15%	5%	99%
Colon cancer and dysautonomia	99%	98%	70%	35%	35%	99%

**Figure 1:**
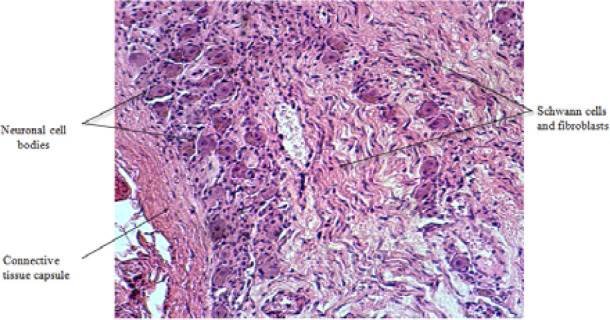
Transverse section of normal celiac ganglion – the nerves are encased in fibrous tissue sheaths that surround the components of the nervous structures

To facilitate comparisons, a normal celiac ganglion tissue sample (H&E, 200×) was included for reference (Figure 1). This allowed a direct comparison between normal tissue, dysautonomia, and colon cancer samples.

**Figure 2:**
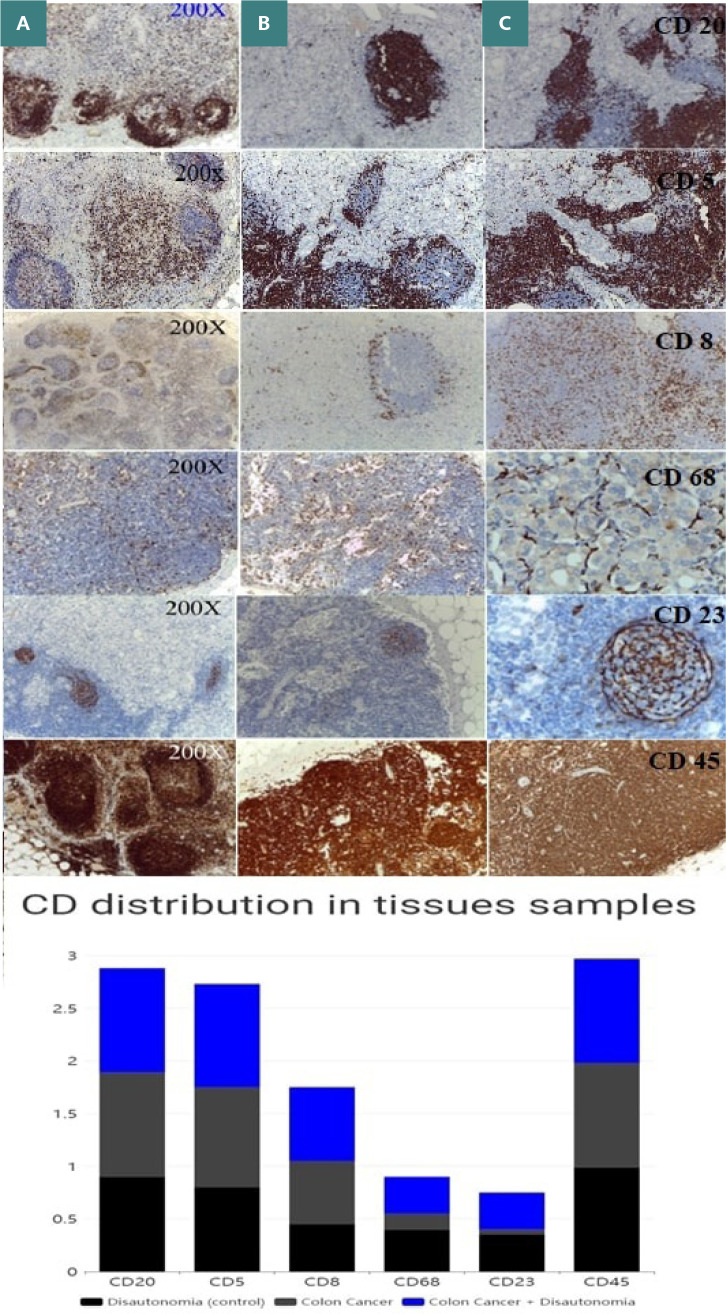
CD20, CD5, CD8, CD68, CD23, and CD45 (brown) immunohistochemical staining of celiac ganglion tissue samples from patients with: A) dysautonomia, B) colon cancer with dysautonomia, and C) colon cancer without dysautonomia. Magnification: 200x. A chart detailing the distribution of CD markers in celiac ganglion samples is also provided.

Images were captured for all celiac ganglion samples. A representative image was selected for each study group and each CD antibody to illustrate the common staining patterns observed across the samples (Figure 2A-C).

The celiac ganglion section labeled for CD20 indicates immune inflammation within the sympathetic ganglion, commonly observed in dysautonomia and colon cancer. CD5 staining revealed focal positivity in lymphoid cells, with scattered macrophages and histiocytes, suggesting an inflammation of immune origin within the sympathetic ganglion. CD8 expression showed a lymphocytic infiltrate consistent with immune-mediated inflammation within the ganglion. The presence of CD8+ subsets suggests activation and recruitment of T cells to the site of inflammation. CD68 staining indicates the presence of macrophages and histiocytes, meaning reactive processes within the celiac ganglion, likely related to inflammation in the case of dysautonomia and colon cancer. The celiac ganglion section labeled for CD23 shows activation and migration into the site of inflammation in the celiac ganglion of dendritic cells. Lastly, CD45 staining demonstrates a lymphocytic infiltrate consistent with immune-mediated inflammation within the ganglion.

A descriptive statistical analysis was performed to assess the variability of IHC marker expression among groups. In the control group with dysautonomia, the percentage scores ranged from 5.00% to 99.00%, with a mean of 62.17%, with a standard deviation of 43.10%. For the colon cancer group, scores ranged from 35.00% to 99.00%. The mean percentage was 64.83%, with a standard deviation of 28.04%. In the colon cancer + dysautonomia group, scores ranged from 35.00% to 99.00%, with a mean of 72.67% and a standard deviation of 31.22% (Table 4).

**Table 4:** Analysis of Variance (ANOVA) results for dysautonomia (control) as the dependent variable

Source	Sum of Squares	df	Mean Square	F	Sig.
Regression	9288,833	5	1857,767	.	.
Residual	,000	0	.		
Total	9288,833	5			

a. Dependent Variable: dysautonomia (control) b. Model: (Intercept), colon cancer, colon cancer + dysautonomia

The ANOVA results indicate that the model was significant, as evidenced by the lack of a "Sig." value for the F-statistic. This suggests that the dependent variable, dysautonomia (control), had a significant effect on the independent variables (colon cancer or colon cancer + dysautonomia). These results provide evidence for a significant relationship between at least one of the independent variables and dysautonomia (control). To further explore these associations, we applied Pearson’s correlation test, Kendall’s tau-b, and Spearman’s rho to assess the relationship between dysautonomia (control) and the colon cancer + dysautonomia group. The results of these analyses are summarized in Table 5.

**Table 5:** Correlations between dysautonomia (control) and colon cancer + dysautonomia and colon cancer

			Colon cancer	Colon cancer + dysautonomia	Dysautonomia (control)
Colon cancer	Pearson Correlation		1	,997^**^	,927^**^
	Sig. (2-tailed)			<,001	,008
	*n*		6	6	6
Colon cancer + dysautonomia	Pearson Correlation		,997^**^	1	,927^**^
	Sig. (2-tailed)		<,001		,008
	*n*		6	6	6
Dysautonomia (control)	Pearson Correlation		,927^**^	,927^**^	1
	Sig. (2-tailed)		,008	,008	
	*n*		6	6	6
				**Colon cancer**	**Dysautonomia (control)**
Kendall’s tau_b	Colon cancer	Correlation Coefficient		1,000	,966^**^
		Sig. (2-tailed)		,007	.
		n		6	6
	Dysautonomia (control)	Correlation Coefficient		,966^**^	1,000
		Sig. (2-tailed)		,007	.
		*n*		6	6
Spearman’s rho	Colon cancer	Correlation Coefficient		1,000	,986^**^
		Sig. (2-tailed)		.	<,001
		*n*		6	6
	Dysautonomia (control)	Correlation Coefficient		,986^**^	1,000
		Sig. (2-tailed)		<,001	.
		*n*		6	6

** . Correlation is significant at the 0.01 level (2-tailed)

The correlation between colon cancer and colon cancer + dysautonomia was extremely strong (r = 0.997, *P* < 0.001), indicating a nearly linear relationship between the two variables. The correlation between colon cancer and dysautonomia (control) was also strong (r= 0.927, *P* = 0.008), indicating a significant positive relationship between the two variables. The correlation between colon cancer + dysautonomia and dysautonomia (control) was also strong (r = 0.927, *P*= 0.008), indicating a significant positive relationship between the two variables. The results suggested that there were strong positive relationships between all three variables. This indicated that there was an underlying mechanism that was driving the relationships between these variables.

To further confirm these associations, Kendall’s tau-b and Spearman’s rho were used to assess the correlation between colon cancer and dysautonomia (control). The results remained consistent with Pearson’s correlation.

Using Kendall's tau_b, the correlation between colon cancer and dysautonomia (control) was strong (τ_b = 0.966, *P* = 0.007), indicating a significant relationship between the two variables. Similarly, Spearman's rho showed an extremely strong correlation (ρ = 0.986, *P* < 0.001), indicating that the relationship between the two variables was robust and not just due to chance.

## DISCUSSION

The intricate relationship between dysautonomia, the immune system, and colon cancer progression is a multifaceted area of ongoing research. In the context of colon cancer, the immune system's dynamics are shaped by the interactions of various immune cell subsets, including CD8+ T cells, CD68+ macrophages, CD23+ B cells, and CD45+ leukocytes, with CD20-expressing B cells and CD5-positive T cells playing key modulatory roles in regulating the immune response and influencing tumor progression [8].

The celiac ganglia play a vital role in regulating the function of many effector tissues, such as the gastrointestinal tract [9]. The celiac ganglion has been recognized as a crucial component of the autonomic nervous system's circuitry, which influences the immune system via its noradrenergic projections to the spleen [10-12].

Given this connection, dysautonomia—an autonomic nervous system dysfunction—has been implicated in oncogenesis, tumor formation, and growth [13]. The autonomic nervous system regulates various physiological processes in the body, including inflammation, immune response, and cell proliferation, and modulating the immune response to cancer [14].

Several studies have suggested a potential link between dysautonomia and oncogenesis in various types of cancer, including colon cancer. Dysautonomia may influence tumor microenvironment, immune responses, and angiogenesis, which are important factors in tumor growth and metastasis [14]. Dysautonomia may be more prevalent in cancer patients, or cancer treatments can trigger autonomic dysfunction, highlighting the complex interplay between the two conditions.

Studies have demonstrated that patients with colon cancer and dysautonomia exhibit altered immune cell populations, including decreased T-cell activation and increased regulatory T-cell activity [15]. This immunosuppressive environment can facilitate tumor growth and metastasis. Alterations in the gut microbiome, common in dysautonomia, can contribute to the development of chronic inflammation and oxidative stress, promoting tumorigenesis [16].

Furthermore, recent studies have highlighted the potential therapeutic benefits of targeting the autonomic nervous system in colon cancer treatment. For example, beta-blockers, commonly used to treat hypertension, have been shown to inhibit colon cancer cell proliferation and induce apoptosis [17].

Building on these findings, this prospective cohort study was designed to investigate the interplay between dysautonomia, colon cancer, and the immune system over one year involving 18 patients. While the very small sample size of this prospective cohort study comprising only 18 patients limits generalizability, such studies play a crucial role in the research landscape by providing early insights and identifying trends that may warrant further investigation. They can highlight unforeseen associations, generate hypotheses, and inform larger, more expansive studies, thus providing a valuable starting point for deeper exploration into the condition. Additionally, a small study can facilitate rapid knowledge accumulation in emerging fields or under-researched areas, ensuring that insights are not overlooked while waiting for larger-scale studies. Our leading question was if there are any correlations between colon cancer and dysautonomia and if one interferes with the other through the immune system.

We used six types of IHC markers in order to assess the results of the study. The use of six specific IHC markers—CD20, CD5, CD8, CD68, CD23, and CD45—is based on their established roles in lymphocyte differentiation, tumor microenvironment characterization, and specific disease pathologies relevant to the study's focus. Anti-CD 20 antibody reacts with a membrane antigen of B-cells [18]. Anti-CD 5 antibody is a T-cell marker specific for neoplastic B-cells in mantle cell lymphoma, chronic lymphocytic leukemia, large B-cell lymphoma, and small lymphocytic lymphoma, and it is useful for diagnosing mature T-cell neoplasm [19].

Anti-CD8 antibody is a useful marker for distinguishing helper/inducer T lymphocytes and is expressed in neoplasms like T-lymphoblastic leukemia and lymphoma [20].

Anti-CD68 antibody marks cells of monocyte-macrophage lineage and detects an epitope associated with lysosomal granules; therefore, lysosome-rich cells stain. This antibody identifies monocytes and granulocytes and is useful in differentiating myelomonocytic and histiocytic tumors [21].

Anti-CD 23 antibody is a B-cell antibody useful for recognizing dendritic cell networks. It also differentiates between small lymphocytic lymphoma, chronic lymphocytic leukemia (CD23 reactive), mantle cell lymphoma, and follicular lymphoma [22].

Anti CD45 is a highly specific antibody for lymphoid or myeloid origin, and it is essential in immune cell activation and differentiation. It is expressed almost exclusively by hematopoietic lineage cells and is present in benign and malignant lymphocytes [23]. Dysautonomia, characterized by dysfunction of the autonomic nervous system, has been observed in patients with various malignancies, including colon cancer. Its presence can indicate severe disease or complications, as it may arise from tumor invasiveness affecting nerve pathways or due to systemic effects of cancer impacting autonomic control [24]. Additionally, dysautonomia may correlate with other manifestations, such as pain syndromes, potentially indicating advanced disease stages. Studies have suggested that autonomic nervous system alterations can predict patient prognosis [25]. This association underscores the complexity of the relationship between dysautonomia and colon cancer, suggesting that disturbances in autonomic function could reflect a broader pathological process influenced by immune responses and tumor behavior. The immunological aspect, particularly the role of immune-mediated inflammation in the celiac ganglia, highlights a potential mechanism linking these two conditions. A primary limitation of this study is the small sample size, with only 18 patients included. Additionally, the total number of celiac ganglia samples analyzed was limited to 108, with each patient contributing six CD antibody-stained samples, which may restrict the generalizability of the findings.

## CONCLUSION

There could be a common pathway between dysautonomia and colon cancer, likely mediated by the immune-mediated inflammation and induced immunosuppressive environment within the celiac ganglion. Regardless of their origin (dysautonomia, colon cancer, or both), tissue samples from the celiac ganglia consistently exhibited positive IHC staining for six different CD markers, though in varying proportions. This supports the notion that the immune system plays a significant role in dysautonomia, whether associated with colon cancer or not, and that immune activity is prominently expressed in the celiac ganglia of individuals with colon cancer, dysautonomia, or both conditions combined. Based on these findings, our study highlights the possibility of immune-mediated inflammation within the celiac ganglion common in dysautonomia and colon cancer. We employed multiple statistical approaches to explore this correlation, including Pearson’s correlation test, ANOVA, Spearman’s rho, and Kendall’s tau-b. These analyses revealed statistically significant associations, reinforcing the hypothesis that dysautonomia and colon cancer are interconnected through immune mechanisms.
